# Enhanced extracellular matrix remodeling due to embedded spheroid fluidization

**DOI:** 10.1088/1367-2630/ade81e

**Published:** 2025-07-10

**Authors:** Tao Zhang, Shabeeb Ameen, Sounok Ghosh, Kyungeun Kim, Mrinal Pandey, Brian C H Cheung, Minh Thanh, Alison E Patteson, Mingming Wu, J M Schwarz

**Affiliations:** 1 School of Chemistry and Chemical Engineering, Shanghai Jiao Tong University, Shanghai 200240, People’s Republic of China; 2 Department of Physics and BioInspired Institute, Syracuse, Syracuse University, Syracuse, NY 13244, United States of America; 3 Department of Biological and Environmental Engineering, Cornell University, Ithaca, NY 14853, United States of America; 4 Fred Hutchinson Cancer Center, Seattle, WA 98109, United States of America; 5 Indian Creek Farm, Ithaca, NY 14850, United States of America

**Keywords:** embedded spheroid, biophysical computational modeling, multicellular interactions, cell–extracellular matrix interactions

## Abstract

Embedding a collective of tumor cells, i.e. a tumor spheroid, in a fibrous environment, such as a collagen network, provides an essential *in vitro* platform to investigate the biophysical mechanisms of tumor invasion. To predict new mechanisms, we develop a three-dimensional computational model of an embedded spheroid using a vertex model, with cells represented as deformable polyhedrons, mechanically coupled to a fiber network via active linker springs. As the linker springs actively contract, the fiber network remodels. As we tune the rheology of the spheroid and the fiber network stiffness, we find that both factors affect the remodeling of the fiber network with fluid-like spheroids densifying and radially realigning the fiber network more on average than solid-like spheroids but only for a range of intermediate fiber network stiffnesses. Our predictions are supported by experimental studies comparing non-tumorigenic MCF10A spheroids and malignant MDA-MB-231 spheroids embedded in collagen networks. The spheroid rheology-dependent effects are the result of cellular motility generating spheroid shape fluctuations. These shape fluctuations lead to emergent feedback between the spheroid and the fiber network to further remodel the fiber network. This emergent feedback occurs only at intermediate fiber network stiffness since at low fiber network stiffness, the mechanical response of the coupled system is dominated by the spheroid and for high fiber network stiffness, the mechanical response is dominated by the fiber network. We are therefore able to quantify the regime of optimal spheroid-fiber network mechanical reciprocity. Our results uncover intricate morphological-mechanical interplay between an embedded spheroid and its surrounding fiber network with both spheroid contractile strength *and* spheroid shape fluctuations playing important roles in the pre-invasion stages of tumor invasion.

## 1. Introduction

Cancer, in its diversity of forms, is a complex phenomenon. It is therefore fitting to deconstruct it into pieces, understand those pieces, and then put the pieces back together again, keeping in mind that strong interactions between the pieces can yield unexpected behaviors. One way to deconstruct cancer is to focus on *in vitro* models. A key *in vitro* model system for studying the combined effects of cell–cell and cell–extracellular matrix (ECM) interactions in a cancer-like setting is an embedded spheroid [1, 2]. More specifically, a spheroid consisting of cancerous cells is surrounded by a collagen network. One of the major goals for studying such a system is to be able to predict whether or not the tumor cells will invade the surrounding collagen. It has been shown that stiffer collagen fibers and collagen density influence tumor invasion [3] and that the viscoelastic relaxation time of the matrix is also a determinant [4]. Moreover, tumor spheroids under perfusion demonstrate that interstitial flow can also affect tumor invasion as can mechanical compression [5, 6]. Tumor spheroids also demonstrate the capacity to radially reorient collagen fibers up to a distance five times the spheroid radius [7] and generate contractile forces on the collagen fibers to remodel it accordingly [8]. In terms of good predictors of tumor invasion potential, some have argued that cell shapes in the spheroid, as opposed to two-dimensional cell migration assays, may be a good predictor [9].

Despite the recent increase in experimental work on embedded spheroids, many theoretical and numerical models for cells interacting with the ECM have focused on single cells, potentially missing important features associated with collective cell behavior [10–14]. And yet, models for bulk tissue and collagen networks abound. One such bulk tissue model is a cell-based, vertex model [15–19], which predicts a density-independent rigidity transition in disordered confluent tissues and micro-demixing in tissue mixtures [17, 20]. These predictions have been verified experimentally [20–22]. As for modeling the ECM, collagen networks are well-approximated by a spring network model containing energetic costs to stretching and bending, otherwise known as fiber networks [23–25]. Strain-stiffening in under-constrained fiber networks has been predicted at finite strain and has been experimentally confirmed [24, 25].

Given the successes of the vertex model and the fiber network model, a natural next step to approximate an embedded spheroid is to couple the two models. In fact, there exists prior work in that direction in two dimensions using a vertex model with interfacial tension at the boundary of a spheroid that couples to a stretchable, three-fold coordinated spring network [26]. Detailed analysis of this two-dimensional model found two regimes with different global spheroid shapes resulting from competition between interfacial tension and tension in the network. In the interfacial tension-dominated regime, the spheroid remains compact with compression-induced fluidization. Interestingly, compression-induced fluidization has also been predicted by others studying the rheology of a similar two-dimensional vertex model in response to oscillatory shear [27]. In the spring network tension-dominated regime, a cavitation-like instability leads to the emergence of gaps at the spheroid-spring network interface to minimize the energy of the coupled system via cell rearrangements at the boundary. Both the compression-induced fluidization and the cavitation-instability are experimentally-testable predictions. The latter prediction may require inhibiting the pathway for cells to make ECM. Moreover, the experimental finding that cells fluidize when surrounded by cancer-associated fibroblasts is consistent with the compression-induced fluidization prediction [28]. As for how does the spheroid affect the mechanics of the spring network, one finds that the location of the spring network’s rigidity transition is sensitive to the spheroid size, mechanical properties and surface tension [29].

While the two-dimensional embedded spheroid model indeed provides insights into the richness of the system, one wonders about the mechanical interplay between the spheroid and the ECM when the spring network is a fiber network with bending stiffness and when there are explicit interactions between the cells and the ECM. To be specific, what happens when there is an extra degree of freedom coupling the cell to the collagen fibers with a molecular clutch-like mechanism as has been determined in single cells coupling to the ECM [30]. Moreover, one wonders how a three-dimensional coupled model would behave as it is not necessarily obvious that one cross-section of a spheroid behaves similarly as another cross-section. In addition, in three-dimensions, there is work coupling an elastic spheroid to a fiber network to very importantly measure forces transmitted in the fiber network [31]. However, there is no cellular-based resolution and not all spheroids are purely elastic. Our new computational model addresses these issues.

Here we couple, for the first time to our knowledge in three dimensions, a cell-based vertex model to a surrounding fiber network model with surface tension at the interface via active linker springs in three dimensions. These active linker springs contract the fiber network by decreasing their rest length as a function of time once they attach. We ask the questions: *Given that tissues can toggle between solid-like behavior and fluid-like behavior, can such changes in spheroid rheology affect the remodeling of the collagen network even before cells start to invade? And how is the collagen remodeling affected by collagen density? And since one anticipates that for low collagen densities the mechanics of the coupled system is dominated by the spheroid and for high collagen densities, the mechanics of the coupled system is dominated by the fiber network, can we identify a regime where both the spheroid and fiber network are on similar footing, mechanically speaking, to optimize mechanical reciprocity between the two?* To begin to answer these questions, we explore how the fiber network remodels for different parts of the parameter space of the model. Moreover, very importantly, we test the predictions of our model against experiments to assess the validity of our computational model.

## 2. An embedded spheroid as a 3D vertex model coupled to a 3D fiber network

### 2.1. Summary

We have developed a three-dimensional computational model for an embedded spheroid. A three-dimensional vertex model represents the cells in the spheroid as deformable polyhedrons with shared faces. The vertex model energy is defined by a very stiff volume spring, a less stiff surface area spring, and a surface tension *γ* for cell faces on the boundary of the spheroid [19]. The cells can exchange neighbors via a reconnection event by replacing an edge smaller than some threshold length with a face or vice versa [18, 19]. A disordered network of crosslinked fibers is modeled on a face-centered cubic (FCC) lattice with randomly diluted bonds with probability $1-p$. Each bond is characterized by an extensional spring constant and two neighboring bonds along one of the principle axes of the FCC lattice are assigned a bending stiffness whose target angle is 180^∘^ [32, 33]. The spheroid consisting of $N_\mathrm{c}$ cells is coupled to the fiber network with $N_\mathrm{f}$ nodes using active linker springs that connect fiber nodes to the center of some fraction of boundary cell faces. The fraction is set by the number of active linker springs, which is kept fixed throughout the simulation, though active linker springs can appear and disappear depending on whether or not a boundary cell face leaves the boundary via a reconnection event. The linker springs actively contract as the target linker spring length decreases at some prescribed strain rate.

**Figure 1.**
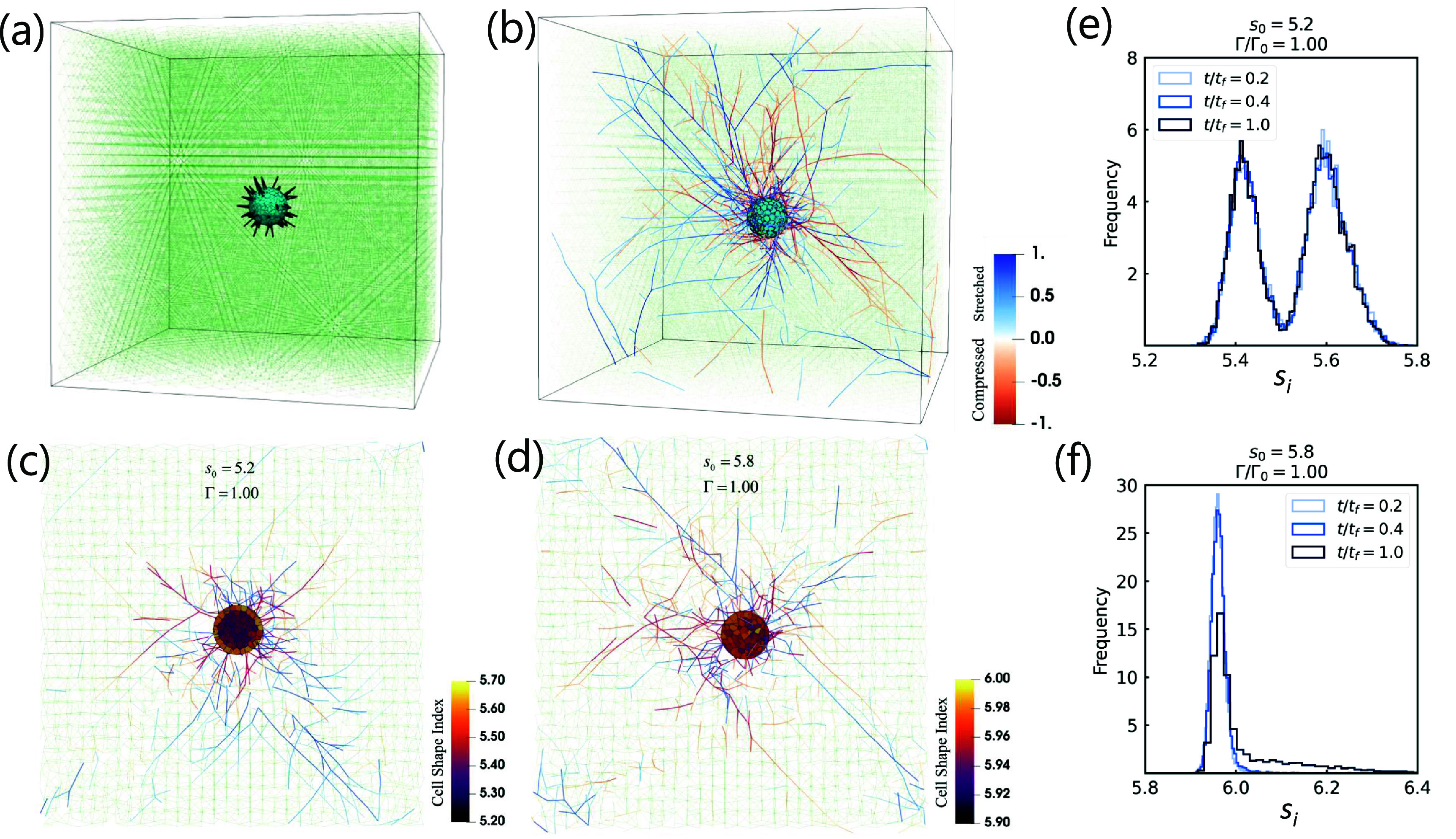
*A 3D computational model for a cell-based spheroid embedded in extracellular matrix*. (a) Initial simulation configuration in which the cells making up the spheroid are shown in cyan polyhedrons, collagen fibers are shown with thin, light green lines and initially occupy a FCC lattice, and the active linker springs connecting the spheroid to the fiber network are denoted with thick, black lines. The equilibrium length of the linker springs decreases with time to capture the action of focal adhesions. (b) The embedded spheroid system at the final time of the simulation. The color scale of the collagen fibers denotes the normalized tension $\tau/\tau_0$ in the fibers with blue indicating extension and red indicating compression and *τ*_0_ denoting the square root of the variance in the tension. (c) Cross-section with cell shape index color map and fiber tension for $s_0 = 5.2$ and $\Gamma/\Gamma_0 = 1.0$ (with $\Gamma_0 = K_V V_0^{4/3}$) and $t/t_\mathrm{f} = 0.8$. (d) Same image as (c) but with $s_0 = 5.8$. (e) Cell shape index (*s_i_*) histogram for solid-like spheroids ($s_0 = 5.2$) at different time points in the simulations. The two-peaked individual cell shape index distribution indicates two different populations of cells, higher cell shape index cells at the boundary and smaller cell shape index cells in the bulk. There is little change in the histogram over the time course of the simulation. (f) Cell shape index histogram for fluid-like spheroids ($s_0 = 5.8$) at the same time points in the simulations as in (e). This histogram changes with time more so in comparison to (f). For all figures, the fiber edge probability is *p* = 0.8.

The energies for the vertex model, the fiber network, and the active linker springs are converted to forces. As for the dynamics, overdamped Brownian dynamics is implemented for the nodes associated with the vertex model via the Euler–Murayama integration method. For the fiber network nodes, the Euler integration method is implemented. Both the vertex model nodes and the fiber network nodes are updated until time $t_\mathrm{f}$. See figures 1(a) and (b) for the initial and final configurations of the embedded spheroid. Please see table 1 for a list of parameters and here now are more details.

**Table 1.** Table of the dimensionless parameters used in the simulations.

Quantity	Symbol	Value
Simulation timestep	$\frac{\mathrm{d}t}{t_0}$	0.005
Simulation time in total	$\frac{t_\mathrm{f}}{t_0}$	25000
Cell area stiffness	$\frac{K_A}{K_V\,V_0^{2/3}}$	0.01
Cell target surface area	*s*_0_	5.2–5.8
Boundary interfacial tension	$\frac{\Gamma}{K_V\,V_0^{4/3}}$	0.25,1.0
Reconnect. Event threshold edge length	$\frac{l_\mathrm{th}}{V_0^{1/3}}$	0.02
Damping	$\frac{K_V\,V_0^{4/3}\,t_0}{\mu}$	1
Active force fluctuation energy	$\frac{k_\mathrm{B}T_\mathrm{eff}}{K_V\,V_0^2}$	10^−4^
Individual fiber bending stiffness	$\frac{K_\mathrm{B}}{K_S l^{^{\prime}}_{0}(0)^{2}}$	10^−4^
Individual fiber diameter	$\frac{d_f}{l^{^{\prime}}_0(0)}$	0.02
Fiber network pore size	$\frac{\xi}{l^{^{\prime}}_0(0)}$	1.0
Edge occupation probability for fiber network	*p*	0.75–0.95
Active linker spring stiffness	$\frac{K_L}{K_S \,l_0(0)^{^{\prime} 2}}$	1.0
Final active linker spring target length	$\frac{l^{^{\prime}}_{t0}}{l^{^{\prime}}_0(0)}$	0.2
Number of spheroid cells	*N*	400
Number of active linker springs	$N_\mathrm{L}$	100
Number of fiber network nodes	$N_\mathrm{f}$	1000
Number of realizations	$N_\mathrm{R}$	20

### 2.2. A vertex model for the spheroid

The biomechanical properties of the spheroid are described by the energy functional:

\begin{align*} E_\mathrm{VM} = K_V\sum_{j}\left(V_{j} - V_{0}\right)^2+K_A\sum_{j}\left(A_{j} -A_{0}\right)^2 +\Gamma\sum_{\alpha}\delta_{\alpha,B} S_{\alpha},\end{align*}

where *A*_*j*_ and *V*_*j*_ represent the area and volume of the *j*th cell, respectively. The terms *K_V_* and *K_A_* are volume and area stiffness coefficients. The volume term, with target volume *V*_0_, corresponds to the cell’s bulk elasticity, while the area term, with target area *A*_0_, relates to the acto-myosin cortex’s isotropic contractility. A larger *A*_0_ implies reduced isotropic contractility, suggesting that all cell faces are equivalent in this aspect. This contrasts with two-dimensional models, where a larger effective target perimeter *P*_0_ (assuming *A*_0_ = 1) indicates a balance between cell–cell adhesion and contractility. Cell–cell adhesion and contractility are coupled, as demonstrated by changes in contractility upon the disruption of E-cadherin in keratinocytes [20, 34]. However, due to shared edges in the vertex model, direct tuning of cell–cell adhesion is not feasible, necessitating the assumption of isotropic contractility. Reduced isotropic contractility might lead to anisotropic contractility, influenced by factors like stress fibers [35].

When modeling spheroids, it is important to consider their boundaries. To address this, we construct a confluent cellular collective, or a clump of confluent cells, by making a cut-out of the bulk periodic system that contains cells with empty cells beyond the boundary between cells and empty space (see our prior work for more details [19]). The cut-out is obtained by keeping every Voronoi cell whose centroid sits closer than a prescribed radius to the origin. Although each retained polyhedron is irregular, their centroids fill a volume whose envelope is spherical on the scale of many cells. Two concentric layers of the discarded Voronoi cells are kept and relabeled empty. A real cell that shares at least one polygon with an empty cell is marked as a boundary cell. Polygons that separate a real cell from an empty one are flagged as interface polygons and carry the interfacial tension Γ. Boundary cells retain all their original polygonal facets; no truncation or smoothing is applied. Empty cells do not contribute mechanical forces, but they are fully permitted to take part in reconnection events. This additional topological freedom allows (i) a surface cell to migrate into the interior and (ii) an interior cell to reach the surface via reconnection events. In practice, reconnections proceed smoothly up to the interface under this rule. *S*_*α*_ indexes the area of boundary cell interface with the empty space. The Kronecker delta $\delta_{\alpha,B}$ equals 1 for boundary faces and 0 otherwise.

Lengths in the model are nondimensionalized with $l = V_0^{1/3}$. Time in the model is nondimensionalized with *t*_0_. The dimensionless shape index $s_{0} = A_{0}/(V_{0})^{2/3}$ is a key parameter. For instance, a regular tetrahedron has $s_0\approx 7.2$.

### 2.3. Fiber network model for the collagen matrix

To create a disordered network of crosslinked fibers, we first occupy a FCC lattice with bonds. Each bond is assigned an extensional spring constant denoted as *K*_S_. Additionally, every pair of neighboring bonds aligned by 180^∘^ is endowed with a bending modulus *K*_B_. To introduce a finite fiber length *L* within this framework, we randomly and independently dilute each bond in the lattice with probability $1-p$. In other words, if *p* = 1, then the FCC lattice is fully occupied with bonds. At the junctures where two fibers intersect, a freely-rotating crosslink is established. The crosslink prevents the fibers from sliding relative to each other, thereby maintaining the structural integrity of the network. However, since the FCC lattice contains twelve nearest neighbors, or six fibers, to crosslink only two fibers, one fiber network node is broken up into 3 separate phantom nodes such that not more than two fibers are crosslinked [24]. Although the different pairs of cross-links may overlap geometrically, they do not constrain each other.

Given these ingredients, the energy functional of the fiber network is

\begin{align*} E_\mathrm{FB} &amp; = \frac{K_{\text{S}}}{2}\sum_{ < ij > } f_{ij}\;\left(l_{ij}-l_0\right)^2\end{align*}

\begin{align*} &amp; \quad + \frac{K_{\text{B}}}{2}\sum_{m = 1}^{3}\sum_{ < ijk > = \pi_{0}}f_{ij}f_{jk}\;\left(\theta_{ijk}^m-\pi\right)^2,\end{align*}

where $f_{ij} = 1$ if a bond is occupied and $f_{ij} = 0$ if not, *l*_*ij*_ represents the length of each bond, $\sum_{\langle ij \rangle}$ represents sum over all nearest neighbor bonds, $\theta_{ijk}^m$ represents the angle between nearest neighbor bonds that are crosslinked by the same phantom node with phantom node index *m*. Moreover, $\sum_{\langle ijk \rangle = \pi_0}$ represents sum over pairs of nearest neighbor bonds sharing a node and only for those aligned along of the principle axes of the initial FCC lattice. The first term in *E*_*FN*_ corresponds to the energy cost of extension or compression of the bonds, while the second term to the energy penalty for the bending of fibers segments made of the three possible pairs (*m* = 1–3) of adjacent collinear bonds forming whose initial angle is 180^∘^. Note that there is no energy cost to fiber twisting.

### 2.4. Embedded spheroid as a vertex model coupled to a fiber network

The spheroid consists of $N_\mathrm{c}$ total cells, while the fiber network comprises $N_\mathrm{f}$ nodes. To integrate these two systems, we establish a coupling mechanism. There are $N_\mathrm{LS}$ linker springs with each connecting a fiber network node to the nearest boundary spheroid cell’s surface polygon center. The equilibrium length of each linker spring *i*, $l^{^{\prime}}_{0i}$, is time-dependent, decreasing linearly at a constant strain rate until reaching a minimum equilibrium length, $l^{^{\prime}}_\mathrm{0min}$. This time dependence captures the molecular clutch framework to focal adhesions in which integrins attach to the ECM fibers [30]. Since the focal adhesion is coupled to the acto-myosin cortex, contractile activity emerges. Changing equilibrium spring lengths has been used previously to encode acto-myosin contractility and so we invoke it here in the form of a decreasing equilibrium spring length [36, 37]. As one boundary cell cannot have more than one linker spring, there is an upper bound on $N_\mathrm{LS}$. For the parameters we are working with generally about 50 percent of the boundary, or surface cells, contain active linker springs. Given these assumptions, the total energy of the active linker springs is quantified by:

\begin{equation*} E_\mathrm{LS} = \frac{K_\mathrm{LS}}{2}\sum_{i = 1}^{N_\mathrm{ls}} \left(l^{^{\prime}}_i-l^{^{\prime}}_{0i}\left(t\right)\right)^2,\end{equation*}

where $K_\mathrm{LS}$ represents the stiffness of each linker spring. Initially, $l^{^{\prime}}_{0i}$ is set at $1.5\,V_0^{1/3}$, which is approximately the spacing of the initial FCC lattice. At simulation time $t_\mathrm{on} = 500t_0$ the rest length $l^{^{\prime}}_{0i}$ of every active linker begins to shorten linearly from $1.5\,V_0^{1/3}$ to $0.2\,V_0^{1/3}$ over time interval $5000\,t_0$. Linkers that form after $t_\mathrm{on}$ start with the current value of $l^{^{\prime}}_{0i}$ and hence $l^{^{\prime}}_{0i}$ does not return to the initial $1.5\,V_0^{1/3}$. The active linker spring is, therefore, dynamic over this time after which time it is not dynamic, unless the active linker spring is removed or created if the cell with an active linker spring moves into the bulk of the spheroid and a new cell moves to the boundary.

If a boundary cell undergoes a reconnection event to become an interior cell, its associated active linker spring is removed. If an active linker spring is removed, another one is created so that $N_\mathrm{LS}$ is a conserved quantity. The new active linker will be chosen from the boundary cells that do not yet contain one. In addition, if the surface polygon of a boundary cell shrinks to an area less than 0.1 or deforms into a concave shape where the polygon center lies on a polygon edge, and the associated active linker spring is consequently removed. These processes for the creation and removal of active linker springs ensure continuous adaptation and restructuring of the linker springs in response to the evolving configuration of the embedded spheroid.

### 2.5. Embedded spheroid system

Finally, we arrive at the total energy for the embedded spheroid system is given by:

\begin{equation*} E_\mathrm{ES} = E_\mathrm{VM}+E_\mathrm{FB}+E_\mathrm{LS}.\end{equation*}

To study this energy functional, we will use a dynamical approach, as opposed to energy minimization, which is detailed next.

### 2.6. Dynamics

Cell dynamics are integral to the model. Indeed, cells are capable of moving past each other while maintaining the confluence of the tissue. In two dimensions, such movements are termed T1 events, and they contribute to understanding the rigidity transition [17]. In three dimensions, these movements are referred to as reconnection events [18, 19]. Prior work has developed an algorithm for such reconnection events focusing on edges becoming triangles and vice versa that can occur for edges below a threshold length $l_\mathrm{th}$, or more precisely, if the maximum length of the edges involved in the reconnection event becomes shorter than the threshold length [18]. There are additional conditions that must be met to ensure that the reconnection event is physically plausible, namely, that the event is geometrically, energetically, and topologically reversible [18]. For instance, to ensure topologically reversibility, the constraint that no two polyhedral cells can simultaneously share two or more polygonal faces was recently added to the list of conditions [19]. Other types of reconnection events may be possible to explore using the graph vertex model where the topology of the network is stored in a knowledge graph [38].

Beyond reconnection events, the model incorporates overdamped Brownian dynamics for each cell vertex. The equation of motion governing the position **r**_*I*_ of a cell vertex *I* is expressed as:

\begin{equation*} \dot{\mathbf{r}}_{I} = \mu \mathbf{F}_{I} + \mu \mathbf{F}_{I}^B,\end{equation*}

where **F**_*I*_ represents the conservative force and $\mathbf{F}_{I}^B$ the random force acting on the $I_\mathrm{th}$ vertex. The conservative force **F**_*I*_ arises from area and volume constraints, encompassing cell–cell interactions, as well as the interfacial tension between the spheroid and the fiber network and the active linker spring interaction. Furthermore, each cell vertex *I* undergoes random fluctuations as encoded in an effective diffusion coefficient $\mu k_\mathrm{B} T_\mathrm{eff}$, with $T_\mathrm{eff}$ denoting an effective temperature. This effective temperature captures the force fluctuations due to the internal, active mechanisms of a cell and is much smaller than the conservative force contribution. Alternatively, one can interpret these fluctuations as a means to probe the complex energy landscape of the conservative forces. Unless otherwise specified, the mobility *µ* = 1. The positional updates for each cellular vertex in the model are executed using the Euler–Murayama integration method.

As for the nodes of the fiber network, they are updated using Euler’s method, based on the forces acting on them due to the other fibers and the active linker springs. We employ overdamped dynamics. There is no active force fluctuation contribution to the fiber network nodes.

A spheroid is then created from a bulk, random Voronoi tessellation from which cells are cut out from. Similarly, edges of the fiber network are removed from the center so that the spheroid can be inserted. The active linker springs are then determined (for a given fraction of boundary faces). After a specific number of time steps, the linker springs begin to contract to their smaller target spring length. Figure 1 shows an initial configuration of the simulation and a final configuration of the simulation. For comprehensive details on the parameters employed in our simulations, including their specific values, refer to table 1. To study this coupled system, the parameters are chosen based on prior simulations of fiber networks and on computational efficiency. To convert simulation units to biophysical units, one simulation length unit $V_0^{1/3}$ is approximately 10 microns, and one simulation time unit *t*_0_ is approximately 0.144 s (the simulation time in total $t_\mathrm{f} = 25000t_0$ is approximately 1 h), while one simulation force unit is approximately one nanoNewton. We have chosen to plot quantities in their dimensionless form. While there are a number of aspects of this computational model to be explored, here we will study the system as a function of the target shape index *s*_0_ and the occupation probability *p*.

## 3. Results

### 3.1. Boundary-bulk effect in cell shape index in solid-like spheroids

Figures 1(c) and (d) shows cross-sectional snapshots of the cell shape index and accompanying linker springs and fiber networks at $t/t_\mathrm{f} = 0.8$ for two different *s*_0_s. For $s_0 = 5.2$, we find that the cells near the boundary of the spheroid have a larger shape index than the cells in the core. We do not readily observe such a trend for the higher $s_0 = 5.8$ case. Given this marked difference in cell shape index for $s_0 = 5.2$ and $s_0 = 5.8$ in figures 1(c) and (d), we study the distribution of shape index for each cell, denoted as *s_i_*. In figures 1(e) and (f) we plot the histogram for the cell shape index for two different target cell shape indices, $s_0 = 5.2$ (e) and $s_0 = 5.8$ (f), and at different time points in the simulation. There does not appear to be much change with time in the histogram for $s_0 = 5.2$. The histogram is double-peaked with the peak at the larger cell shape index describing the boundary cells and the peak at the smaller cell shape index characterizing the bulk cells. A similar histogram was observed for a spheroid/organoid studied earlier in the absence of the fiber network [19]. Note that for the more solid-like spheroid, the bulk cells are not able to achieve their target cell shape index as they are not able to rearrange as readily within the spheroid via reconnection events. However, the boundary cells, as they interact with the fiber network do take on larger cell shape indices. For the fluid-like spheroid, on the other hand, the cell shape indices evolve more in time with the histogram becoming more broad, though still dominated by a single peak slightly higher than $s_0 = 5.8$. The broadening occurs for the boundary cells as they interact with the surrounding fiber network. Therefore, one can readily distinguish between the two rheologically different spheroids by looking at the cell shape index distribution.

Interestingly, the shift from a more solid-like spheroid to a more fluid-like spheroid occurs at an $s_0\approx 5.7$, which is higher than the bulk result [19]. This finding is not surprising, given earlier calculations for the two-dimensional case of a spheroid with an interfacial boundary tension where the transition point shifts to due to the interfacial boundary tension [26]. When we explore the difference in cell shape index distributions between the more solid-like and fluid-like spheroids for small interfacial tension, we find the two peaks for the solid-like spheroid start to merge into one as there is less of a distinction between bulk cells and boundary cells. See figure S1. Moreover, the single peak in the fluid-like case is more broad.

In figure S2 we plot the histogram of individual cell volumes nondimensionalized by *V*_0_, or $V_i/V_0$, for $\Gamma/\Gamma_0 = 1.0$ demonstrating that the cell volumes are approximately 4 percent less than the target volume of one for the fluid-like spheroids, while the cell volumes in the solid case differ more so from their target volume in a two-peaked fashion. Indeed, as a cell is not a closed system, its volume is not expected to be perfectly conserved. Earlier work with tumor spheroids indicated the addition of dextran to the environment of a tumor spheroid to exert mechanical stress on it [39]. The volume of cells near the core of the tumor spheroid in response to this applied mechanical stress decreased within minutes [39]. We observe a decrease in the volume of the cells for both the solid-like and fluid-like spheroids.

### 3.2. Cell motion within the spheroid and active linker forces

#### 3.2.1. Cellular rearrangements are more frequent in fluid-like spheroids than solid-like spheroids

**Figure 2.**
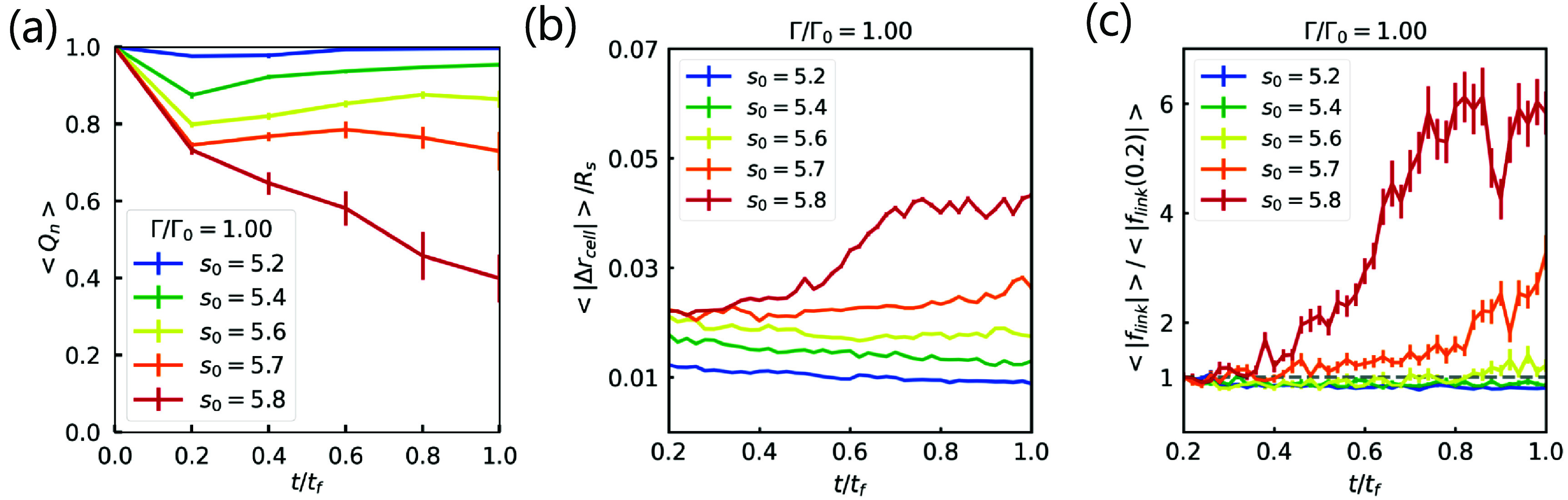
*Characteristics of fluid- versus solid -like spheorids as revealed by the computational model.* Fluid-like spheroids are shown to undergo higher cell neighbor re-arrangement (a), position displacement (b) and higher active linker tension (c). (a) Plot of the average fraction of cells that do not exchange neighbors, $ < Q_n > ,$ as a function of time for different *s*_0_.(b) Plot of the magnitude of the cell displacement $|\Delta r_\mathrm{cell}|$ (nondimensionalized by the spheroid radius $R_\mathrm{s}$) by tracking cell center over a fixed time window as a function of time for different values of the target shape index. Cells displace more in the fluid-like spheroids than in the solid-like spheroids with the difference accentuated for $t/t_\mathrm{f} > 0.6$. (c) Plot of the average linker spring force (nondimensionalized by the average linker force at one simulation time point) as a function of time. The average linker spring force increases at later times more significantly for fluid-like spheroids as compared to solid-like spheroids. For all figures, $\Gamma/\Gamma_0 = 1$ and *p* = 0.8.

We also plot the fraction of cells who have *not* lost two or more of their neighbors on average as a function of time, denoted by $ < Q_n > $ (figure 2(a)). In other words, if no cells exchange neighbors, then $ < Q_n > = 1$, and the spheroid is a solid. However, if $ < Q_n > = 0$, then all cells are performing neighbor exchanges within the spheroid. For smaller *s*_0_ values, we observe that most cells are not finding new neighbors. As the active linker springs contract until $t/t_\mathrm{f} = 0.2$, the fraction of cells rearranging decreases somewhat and then increases to a fraction that is close to unity. These spheroids are more solid-like. However, for $s_0 = 5.7,5.8$, the fraction of cells undergoing neighbor exchanges continues to decrease even after the active linker springs stop contracting. The spheroid is becoming increasingly fluid-like as it interacts with the fiber network. This increasing fluidization could eventually set the stage for tumor invasion.

More cellular rearrangements should lead to enhanced cell motion. We also measure explicitly cell motion by tracking the average displacement of a cell center as a function of time for different target cell shape indices and for two different interfacial tensions (see figures 2(b) and S3). We find that the displacement of the cell centers, beyond the initial displacement due to the initial contraction of the active linker springs, does not increase with time for the more solid-like spheroids, while for more fluid-like spheroids the cell center displacement does increase over time. While we have used the cell shape index distribution to delineate between solid-like and fluid-like spheroids, here we present evidence for such differences in rheology in terms of cell motion. Indeed, there does appear to be a delineation occurring between $s_0 = 5.6$ and $s_0 = 5.7$, as indicated earlier. Prior bulk analysis led to a rigidity transition location at $s_0^* = 5.4$. However, recent two-dimensional analysis of a vertex model coupled to a spring network found that the presence of the interfacial tension did alter the location of the rigidity transition [26]. More specifically, a spheroid with interfacial tension can be mapped to a bulk model with a shift in the dimensionless area and area spring stiffness so that the location of the transition shifts [26]. Our three-dimensional results are consistent with the prior two-dimensional results, particularly as figure S3 shows that for smaller interfacial tension, the delineation between fluid-like and solid-like occurs at a larger *s*_0_.

#### 3.2.2. Forces exerted by the active linker springs are higher for fluid-like spheroids

In figure 2(c) we show the average total active linker spring force as a function of time $t/t_\mathrm{f}$ for different target cell shape indices. For smaller target cell shape indices, after the initial contraction phase of the active linker springs, the average total active linker spring force remains constant as a function of time. However, for the larger target cell shape indices, after approximately $t/t_\mathrm{f} = 0.4$ for $s_0 = 5.8$, for example, the average total active linker spring force begins to increase with time. Figure S4 shows the average linker spring force as a function of time for the smaller interfacial tension case resulting in a smaller active linker spring force on average.

### 3.3. Fiber network remodeling

#### 3.3.1. Fluid-like spheroids densify and radially align the fiber network more than solid-like spheroids in simulations

We observe remodeling of the fiber network for different target shape indices and different fiber network stiffnesses by tuning the fiber edge occupation probability *p*. For the latter property, the larger *p* is the larger the fiber network stiffness. More precisely, the larger the shear modulus of the fiber network [32, 33]. See figure S5 for a plot of the fiber network shear modulus as a function of shear strain for different *p*s (or different average coordination number *Z*).

To better understand how the spheroid remodels the fiber network, we first report on the average of the magnitude of fiber displacement as a function of radial distance from the center of mass of the spheroid in figure S6. The displacement is taken from $t/t_\mathrm{f} = 0$ to $t/t_\mathrm{f} = 1$. We find for all target cell shape indices, *s*_0_ = 5.2–5.8, that the magnitude of the average fiber displacement within concentric shells emanating from the spheroid is larger closer to the spheroid in comparison to further away from it. This trend is consistent for the system sizes we study, which include smaller system sizes than the one shown in figure S6. We also observe that the smaller *s*_0_ spheroids do not displace the fiber network as much as the larger *s*_0_ spheroids. There is a slightly more dramatic difference in the magnitude of displacement for $s_0 = 5.7$ and $s_0 = 5.8$, indicating that the spheroid rheology may differ between $s_0 = 5.6$ and $s_0 = 5.7$, which is consistent with the change in the cell shape index histogram as discussed above.

**Figure 3.**
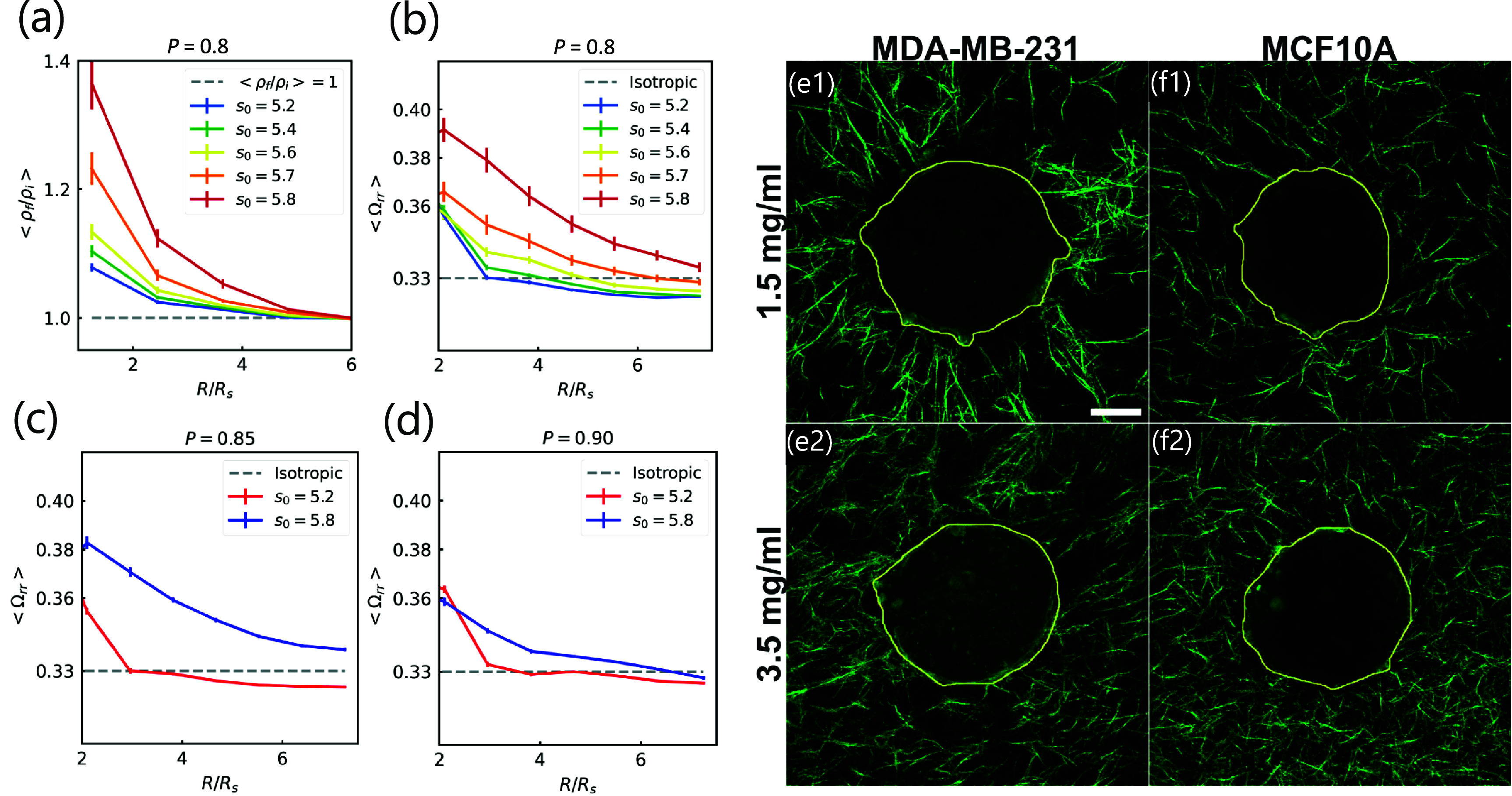
*Fluid-like spheroids align the fiber network more than solid-like spheroids and the extent of the alignment depends on the fiber network stiffness.* (a) Fluid-like spheroids densify fiber network near the spheroid more than solid-like spheroids. The larger the target cell shape index *s*_0_, the more the fibers are densified towards the center of the system. Note that $\rho_\mathrm{i}$ denotes initial fiber density, $\rho_\mathrm{f}$ denotes the final fiber density, and $R_\mathrm{s}$ denotes the radius of the embedded spheroid system. Here, the fiber edge occupation probability, *p* = 0.8. (b) Fluid-like spheroids radially realign the fiber network near the spheroid more than solid-like spheroids. Plot of the radial component of the fiber orientation tensor as a function of radial distance *R* from the center of mass of the spheroid for all fibers. Here also, *p* = 0.8. (c) The radial realignment of the fiber network is less slightly less significant near the spheroid with a higher fiber network stiffness with *p* = 0.85 and in (d) with *p* = 0.9 the realignment of the fiber network is even less significant. Moreover, there is not much difference between the fluid-like spheroid and the solid-like spheroid. All computational results are with a dimensionless interfacial surface tension of $\Gamma/\Gamma_0 = 1$. (e) and (f) experimental results revealing that the collagen fiber network is remodeled differently by malignant (MDA-MB-231) vs non-tumorigenic (MCF-10A) spheroids. Confocal reflectance images of MDA-MB-231 tumor spheroids embedded in 1.5 mg ml^−1^ collagen (e1) and in 3.5 mg ml^−1^ collagen (e2). (f) Reflectance confocal images of MCF10A spheroids embedded in 1.5 mg ml^−1^ collagen (f1) and 3.5 mg ml^−1^ collagen (f2). All images were taken at $t = 2\,\mathrm{h}$, which is 2 h after spheroid seeding and collagen polymerization. The scale bar is 50 *µ*m. Each image is 354.2 × 354.2 *µ*m. Each image is a maximum *z* projection of a 15 *µ*m thick reflectance confocal *z* stack taken at the mid-plane of the spheroids.

In addition to the magnitude of the displacement of the fiber network, as a result of being coupled to the spheroid, one can ask about the direction of the displacement. Given the spherical symmetry of this bi-material system combined with prior experimental observations, we instead focus on the density of the fiber network as a function of the radial distance from the center of mass of the spheroid. Should the density of the fiber network that is closer to the spheroid increase, then the spheroid, with its active linker springs, has radially pulled/contracted the fiber network towards it and vice versa. We find that the fiber network is displaced radially toward the spheroid with an enhancement of fiber density closer to the spheroid in comparison to further away from it (see figure 3(a)). Prior two-dimensional simulations of a cellular Potts model coupled to a fiber network also demonstrate such radial densification behavior [40] as do experiments [31]. Finally, as the more fluid-like spheroid can displace the fiber network more so than the solid-like spheroid, there is more radial densification of the fiber network for the fluid-like spheroids.

Now that we have evidence for the spatial remodeling of the fiber network by the spheroid, let us probe the orientation of the fibers as a result of it by computing the fiber orientation tensor *Ω*_*xy*_. Figure 3(b) shows *Ω*_*rr*_ as a function of radial distance from the center of mass of the spheroid. Should there exist a bias of fiber modeling along the radial direction, then *Ω*_*rr*_ should be larger than $1/3$. We find that for fluid-like spheroids that can remodel the fiber network to a greater extent, closer to the spheroid, the fibers are oriented more radially, while further away they are more uniformly oriented. This computational finding will be qualitatively supported by experiments detailed below and has been found in other types of tumor spheroids [7]. Figure S7, however, demonstrates that for high-tensioned edges, or edges that are strained beyond 0.1%, there is a very high degree of radial ordering for the solid-like spheroids. As the spheroids become more fluid-like, the high-tensioned fibers become less radially oriented. Interestingly, a high degree of radial alignment in high-tensioned fibers was found in earlier work focusing on a radially-contractile monopole in a fiber network in both two and three dimensions [41]. Here, the spheroid has many degrees of freedom, including the linker springs, which makes the problem more complex indeed.

We interpolate between the radially-contracting monopole [41] and our work to briefly study, for simplicity, a triangular lattice of fibers with six active linker springs, which has 13 more degrees of freedom than a contractile monopole, via energy minimization we also observe the same trend of high-tension fibers as more radially aligned and compression fibers being oriented circumferentially near the spheroid. See figure S8. For this simpler two-dimensional model, at full occupation probability, the smaller the final target equilibrium spring length, the larger the average displacement in the fiber network, not surprisingly. For smaller occupation probabilities, this trend is less clear due to the flipping of nodes. Such flipping of nodes does not readily occur in three dimensions. So for a spheroid with a few degrees of freedom, the contractile monopole is a reasonable approximation. It is therefore feasible that the solid-like spheroids better approximate the contractile monopole case as there are far fewer cellular rearrangements and so additional degrees of freedom represent an elastic object that radially contracts the fiber network.

To understand how interfacial tension affects the fiber network remodeling trends in terms of average magnitude of the fiber displacement as a function of distance from the center-of-mass of the spheroid, in figure S6, we plot this quantity for smaller spheroid surface tension. We find similar trends as with the larger spheroid surface tension, though the average magnitude of the fiber displacement is not quite as dramatic as for the larger interfacial tension case as the spheroid is not as effectively strong as a contractile object on the fiber network. To provide additional interpretation, the stronger a contractile force, the more densification of the fiber network around the spheroid. According to a simplified analysis, the contractile force allows one to probe the stretching and bending of the fiber network and to what spatial extent it can do so. To assess the spatial extent of the contractile spheroid, when looking at the average displacement curves, we numerically compute the first derivative to look for a crossover between bending (scaling with $r/R_\mathrm{s}$) and stretching (scaling with $1/(r/R_\mathrm{S})^2$). We find that for $s_0 = 5,2,5.4,5.6$, the slope of the curves approaches a constant approximately near $R/R_\mathrm{s} = 4$. However, for $s_0 = 5.7, 5.8$, the slope does not approach a constant indicating a different effective lengthscale over which the spheroid is acting. More system-size studies will need to be conducted to quantify this length scale, which appears to be larger for more fluid-like spheroids.

We also modify the fiber network stiffness in figures 3(c) and (d) by increasing the fiber edge probability *p* and measure the extent of the radial alignment. As *p* increases from 0.8 to 0.85 to 0.9. For *p* = 0.9, there the difference in the radial fiber alignment between the fluid-like spheroids and the solid-like spheroids is minimal. Moreover, for both spheroid types, the radial fiber alignment is not as significant as it was for *p* = 0.8. For *p* = 0.75, we find similar results as for *p* = 0.9 (see figure S9). In other words, *p* = 0.8 represents the fiber network stiffness where the fiber network remodeling is the most significant. Given the increased constraints in the fiber network due to the crosslinking, it is mechanically more difficult for the spheroid to remodel it and so we anticipate that the mechanics of the system becomes more fiber network dominated. On the other hand, at lower fiber network stiffness, the coupled system mechanics becomes dominated by the spheroid.

We also quantify the strain in the fiber network as a function of time with negative strain denoting compression. In figure S10(left), we observe that after $t/t_\mathrm{f} = 0.2$ for $s_0 = 5.2$, the strain histogram does not evolve with time, noting that at $t/t_\mathrm{f} = 0$ all edges in the fiber network exhibit zero strain. After $t/t_\mathrm{f} = 0.2$, the active linker springs have finished with their contraction and so the system remains somewhat static in terms of forces with few new active linker springs being created or destroyed as their is very little exchange between boundary cells and bulk cells. However, for $s_0 = 5.8$, the strain histogram does evolve with time (see figure S10(right). Also note that an asymmetry in the strain histogram develops for both types of spheroids. The radial alignment of high-strain fibers facilitates higher strain as compared to the negative strain circumferentially-oriented fibers. Furthermore, these high-strain fibers exhibit strain stiffening [24]. For the more fluid-like spheroids, the strain asymmetry becomes even larger with more fiber network remodeling.

#### 3.3.2. Radial fiber network alignment in experiments

Radial fiber alignments are seen to be more pronounced in collagen gel near malignant tumor spheroid (MDA-MB-231) in contrast to that near the non-tumorigenic spheroids (MCF10A) shown by the reflectance confocal images in figures 3(e) and (f). It has been suggested in previous literature that MDA-MB-231 tumor spheroids are more fluid-like than MCF10A spheroids [42]. Therefore, our experimental results are qualitatively consistent with the theoretical prediction discussed below that fluid-like tumor spheroid remodel collagen networks more significantly than solid- like tumor spheroids. Furthermore, the alignment enhancement by malignant tumor spheroid is more evident at low collagen concentration of 1.5 mg ml^−1^ in contrast to the high concentration of 3.5 mg ml^−1^. Our experimental results are also qualitatively consistent with the theoretical prediction discussed below that higher collagen density limits the amount of remodeling by the tumor spheroid.

### 3.4. Spheroid shape index fluctuations and emergent mechanical feedback with the fiber network

**Figure 4.**
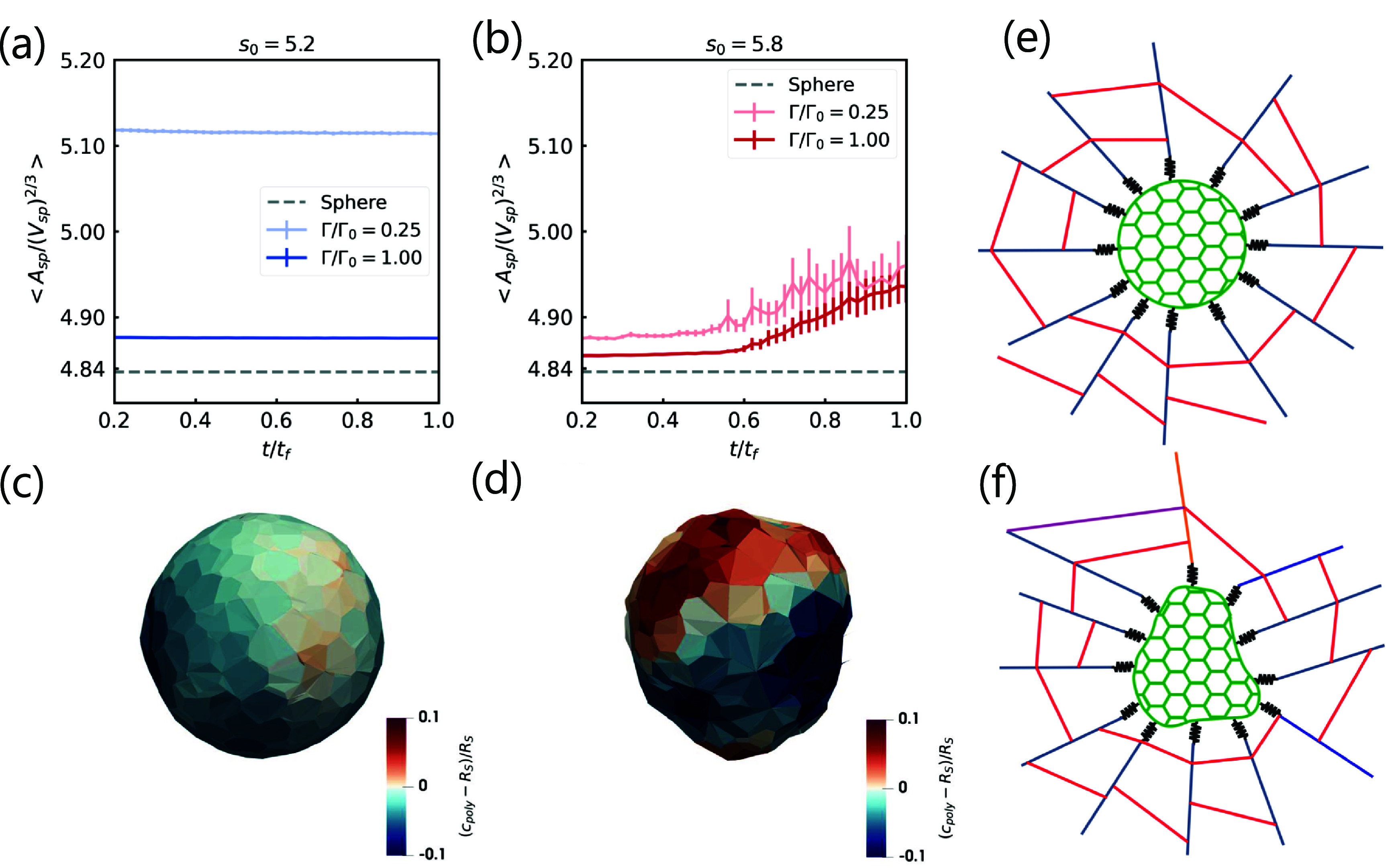
*Positive mechanical feedback between fluid-like spheroids and the fiber network as revealed by simulations.* As the fluid-like spheroids become more fluid-like, the overall spheroid shape distorts to promote further remodeling of the fiber network, which then promotes further fluidization of the spheroid. The amount of feedback depends on the stiffness of the fiber network. (a) Spheroid shape index ($A_\mathrm{sp}/V_\mathrm{sp}^{2/3}$) as a function of time for $s_0 = 5.2$ and for two different values of the dimensional interfacial tension. (b) Same as (a), but for $s_0 = 5.8$. Note that the dashed line in both plots denotes the spheroid shape index of a perfect sphere. (c) The eventual increase in spheroid shape index for fluid-like spheroids corresponds to local deviations from the initial spherical surface. Here, such surface deviations are quantified by the difference between polygon center to spheroid center distance ($c_\mathrm{poly}$), and the initial radius of the spheroid ($R_\mathrm{S}$), i.e. red regions denote outward bulges and blue regions denote inward bulges (or wrinkles). Time of the snapshot is $t/t_\mathrm{f} = 0.4$. (d) Same as (c) but for $t/t_\mathrm{f} = 1.0$. (e), (f) Schematics illustrating how spheroid shape changes can drive fiber network remodeling. If one considers a spheroid as a spherical contractile force monopole with some radius (e), then the fiber network remodeling will typically consist of radial, high-tension ropes with circumferential compressed fibers. However, with spheroid shape changes (in the fluid phase) leading to changes in surface curvature (f), where the spheroid shape bulges outward, the radial, high-tension ropes may be compressed to lead to more intricate remodeling of the fiber network (as compared to a spherical contractile force monopole with some radius). Moreover, spheroidal shape changes break radial symmetry, which then manifests in a less radially oriented fiber network that is prominent in the high-strain fibers. The remodeling of the fiber network by the spheroid can subsequently affect the spheroid to induce additional cellular rearrangements. Thus, the spheroid shape fluctuations create a positive morphology-mechanics feedback loop between the spheroid and the fiber network to ultimately enhance remodeling in both structures. For all computational figures, *p* = 0.8 and $\Gamma/\Gamma_0 = 1.0$.

We now explore in a little more detail how the enhanced remodeling of the fiber network occurs. We have already noted that cellular displacements and active linker forces increase around $t/t_\mathrm{f} = 0.4$ (figures 2(b) and (c)). One consequence is that the cell fluidity leads to changes in spheroid shape with larger fluctuations, as evidenced in figure 4. Indeed, for the more solid-like spheroids, the spheroid shape, defined as $A_\mathrm{sp}/V^{2/3}_\mathrm{sp}$, where $A_\mathrm{sp}$ and $V_\mathrm{sp}$ denote the spheroid surface area and volume respectively, does not change with time, while for the more fluid-like spheroids, the spheroid shape does change with time. Note that the spheroid shape is more spherical for the larger interfacial tension, which is expected. It is the change in spheroid shape that then moves the active linker springs, which continue to remodel the fiber network to displace the fibers. Interestingly, even though there is presumably a lack of bias in the spheroid shape fluctuations, meaning the shape can distort either ‘inwards’ or ‘outwards’, the fluctuations lead to enhanced fiber densification. See figures 4(c) and (d). For spheroid deformations that move inward, the fiber network is strain stiffened. For spheroid deformations that move outward, the fiber network compression softens. Given the asymmetry of the response of strain stiffening and compression softening, the strain stiffening dominates to lead to more overall tension in the network as indicated in the tension/compression distribution of the fiber network.

Given the internal degrees of freedom of the spheroid, it is the spheroid shape fluctuations that give rise to a more complex response than a simple contractile monopole, or even a less simple elastically deformable object. The fluctuations are due to the dynamics/movement of the cells within the spheroid that then modify the interactions between the spheroid and the fiber network by making the system more active in a fluctuation sense to allow for more remodeling of the fiber network, as opposed to the solid-like spheroids where such spheroid shape fluctuations do not occur as readily. With the spheroidal shape distortions comes a breaking of the spheroidal radial symmetry. This breaking of radial symmetry leads to high-tensioned fibers that are less radial symmetric and so explains why the radial alignment of the high-tensioned (or high-strained) fibers decreases with increasing *s*_0_ as the spheroid becomes more fluid-like. Moreover, the spheroid shape fluctuations also lead to additional remodeling of the fiber network, which, in turn, affects the spheroid to lead to further remodeling of the spheroid and so further remodeling of the fiber network. Thus, we find an emergent feedback loop even with this simple model in which there is no explicit feedback between the fluid-like spheroid and the fiber network.

Our computational studies reveal that the strength of a contractile object does not alone determine the remodeling of the fiber network. There is a key additional contribution by the active shape fluctuations due to active cellular rearrangements. As cellular rearrangements are not significant in solid-like spheroids, this emergent feedback between the solid-like spheroid and the fiber network is not a prominent feature. Moreover, as the fiber network stiffness increases or decreases, the emergent feedback is prevalent as the mechanics of this bi-material is dominated by one component or the other making feedback between the two less likely.

## 4. Discussion

We have developed a one-of-a-kind three-dimensional computational model for a spheroid embedded in a fiber network mimicking the ECM. In addition to cellular-level resolution, we incorporate explicit focal adhesion attachment between the cells and the fiber network in the form of an active linker spring whose equilibrium spring length decreases with time. After the initial contraction of the active linker springs, we find that the fluidity of the spheroid can drive spheroid shape changes, which allows for enhanced fiber network modeling as regions of the spheroid that move inward can strain stiffen the fiber network, while regions of the spheroid that move outwards can compression soften the fiber network. Given the force asymmetry between compression softening and strain stiffening, the strain stiffening dominates leading to an asymmetry in the distribution of tension and compression in the fiber network. Moreover, as a fluid-like spheroid distorts itself due to cellular rearrangements, there is additional remodeling of the fiber network, which, in turns, affects the spheroid, to create a positive morphology-mechanics feedback loop that breaks spheroidal radial symmetry. Therefore, counterintuitively, a fluid-like spheroid more readily remodels the fiber network than a solid-like spheroid, at least for an intermediate range of fiber network stiffnesses. So while the strength of a contractile spheroid also impacts fiber network remodeling, we have demonstrated that strength alone, is not the only determinant, as both the fluid-like and solid-like spheroids have the same number and stiffness of active linker springs. See figures 4(e) and (f) for a schematic illustration summarizing this effect. In addition, an increase in spheroid interfacial surface tension compactifies the spheroid and so makes it a stronger contractile puller. However, an increase in interfacial surface tension decreases the spheroid shape fluctuations, thereby decreasing the positive feedback between the spheroid and the fiber network.

Experiments here support the notion that fluid-like spheroids remodeling the fiber network more than solid-like spheroids. Our results here also contribute to understanding how embedded spheroids composed of mouse embryonic fibroblast cells remodel the collagen matrix, while embedded spheroids composed of the vimentin-null mouse embryonic fibroblast cells do not [43]. Other experiments demonstrate that embedded spheroids with cells undergoing coordinated angular motion within the spheroid also remodel the fiber network more so than spheroids with cells not exhibiting such collective motion [44]. Yet, for a direct, quantitative comparison with such experiments, more work is needed on the modeling side in terms of tuning the fiber network’s characteristics to quantitatively match the experiments as well as the shape of pre-invading cells.

How do our results compare with a single-cell interacting with a fiber network? Prior work demonstrates that as cells contract the fiber (collagen) network, there emerge two opposing sides of the cells that dominate [10]. The cell then forms stress fibers between these two opposing sides to lead to elongation of the cell along the polarization direction [10]. As the elongation occurs, presumably there are fewer focal adhesions between the cell and the fiber network in directions orthogonal to the polarization direction, and the cell now mimics more of the fiber network by becoming ‘long and skinny’, i.e. there exists mechanoreciprocity between the cell and the fiber network [45]. When the cells exist in a collective, such as a spheroid, the mechanoreciprocity between an individual cell and the fiber network is not as pronounced, at least in the pre-invasion stages, such that the cellular program, to build stress fibers along a polarization direction does not yet exist. The spheroid can, therefore, manipulate the fiber network in ways that can differ from an individual cell. For instance, the spheroid can still randomly pick out more than one polarization direction, and the shape of the spheroid need not conform to the morphology of the fibers (see figures 4(e) and (f)). The cell–cell reciprocity and the cell-ECM reciprocity thus compete to give a spectrum of possible emergent behavior. We suspect that once cells along the boundary of the spheroid become sufficiently elongated and, therefore, exhibit higher stress, they will be the candidate break-out cells.

Given the richness of our computational model, many questions remain and will be explored in future work. For example, what happens when we incorporate cellular-based forms of explicit, mechanical feedback between the cells and the fiber network, such as cells becoming more contractile the higher the strain in a fiber? This explicit feedback may lead a more heterogeneous distribution of cell shape indices, which could presumably enhance the breakout potential of tumor cells. One can also explore more detailed models of the active linker springs that attach or detach depending on the strain in fiber network. This detail may contribute to understanding the twitching phenomenon observed in embedded spheroids, where the contraction of the fiber network oscillates with time [31], though we already observe hints of strain in the fiber network increasing and decreasing for the fluid-like spheroids. See figure S11. And what happens when viscoelasticity of the collagen network is explicitly included in the model, such as force-sensitive crosslinks, to connect with prior work [4, 46].

Finally, what are the implications of our model for understanding cancer? As fluid-like spheroids can remodel the fiber network to a greater extent than solid-like spheroids, we hypothesize that fluid-like spheroids have more invasive capability, assuming that the remodeling can promote, and not hinder, cell motility. While we leave explicit cell breakout studies for future work, as we see here, much goes into the mechanical crosstalk to help set the stage for potential cell breakout that must be understood. Recent experiments demonstrate that cell breakout is determined by a competition between cortical contractility and stress-fiber contractility [47]. We need such starting points to go beyond the current automaton models [48–50] and include both mechanics and chemical signaling [51] to begin to make quantitative predictions for cell breakout. While cell breakout is the obvious next step, we must also consider the multiscale aspect of cells [52] as well as the adaptability of cells and their ability to ‘train’ the fiber network to be able to escape within a physical learning framework [53–55] just as neural networks are trained to perform a specific function. Moreover, cancer cells interact with other types of cells, such as immune cells, providing an entire cellular ecology as a backdrop with cancer cells trying to train immune cells and vice versa [56, 57]. All such elements will help provide a more accurate, quantitative picture of the complexities of cancer and other diseases and biological processes more generally.

## 5. Methods

### 5.1. Cells, spheroids, and 3D spheroid culture preparation

#### 5.1.1. Cells

Metastatic breast adenocarcinoma MDA-MB-231 cells (ATCC) and non-tumorigenic epithelial MCF10A cells (provided by the Cornell Center for Microenvironment and Metastasis) were used to prepare spheroids. The growth medium for MDA-MB-231 cells consisted of DMEM high glucose medium (Cat. 11965092, Gibco), supplemented with 10% fetal bovine serum (Cat. S11150, Atlanta Biologicals) and 1% antibiotics (100 units ml^−1^ penicillin and 100 *µ*g ml^−1^ streptomycin, Cat. 15140122, Gibco). For MCF10A cells, the medium consisted of DMEM/F-12 (Cat. 11320033, Gibco) supplemented with 5% donor horse serum (Cat. S12150, Atlanta Biologicals), 20 ng ml^−1^ human EGF (Cat. PHG0311, Gibco), 0.5 *µ*g ml^−1^ hydrocortisone (Cat. H0888-1G, Sigma-Aldrich), 100 ng ml^−1^ Cholera Toxin (Cat. C8052-.5MG, Sigma-Aldrich), 10 *µ*g ml^−1^ insulin (Cat. 10516-5ML, Sigma-Aldrich), and 5% antibiotics (Gibco). Both cell lines were cultured for up to 20 passages and used at 50%–70% confluency. DMEM/F12 media was used for spheroid preparation in both cell types [58].

#### 5.1.2. Spheroids

Uniformly sized spheroids were generated using a custom microwell array platform [59, 60]. Silicon masters of 18 × 18 microwell arrays were fabricated in the Cornell Nanofabrication Facility using a one-layer photolithography method. Microwells were patterned onto 1 mm-thick agarose sheets (1 × 1 cm) using soft lithography. Each microwell had a cylindrical shape with a 400 *µ*m diameter and depth. Low adhesion on the agarose gel surface promoted cellular clustering into spheroids. Six microwell arrays were placed in a 12-well plate (Cat. 07-200-82, Corning). Within each well, 3 million cells were suspended in 2.5 ml DMEM/F12 growth medium. Plates were incubated in a 5% CO_2_ environment at 100% humidity for seven days, with medium replenished every 2–3 d. Spheroids were harvested on day 7. Tumor spheroids were filtered using a Falcon Cell Strainer (100 *µ*m pores, Cat. 352360, Corning) to ensure uniform size.

#### 5.1.3. Spheroid-embedded ECM preparation

To create 3D tumor spheroid cultures, spheroids were suspended in 1.5 mg ml^−1^ and 3.5 mg ml^−1^ type I collagen matrices (rat tail tendon, Cat. 354249, Corning). For 1.5 mg ml^−1^ collagen, 27.1 *µ*l collagen stock (11.07 mg ml^−1^) was titrated with 0.6 *µ*l 1 N NaOH and 20 *µ*l 10X M199 (Cat. M0650-100ML, Sigma), adjusted to a final pH of ∼7.4, and diluted with 152.3 *µ*l spheroid suspension in DMEM or DMEM/F12. For 3.5 mg ml^−1^ collagen, 63.23 *µ*l collagen stock was titrated similarly with 1.39 *µ*l 1 N NaOH and 20 *µ*l 10X M199, followed by 115.37 *µ*l spheroid suspension. Each preparation yielded a final volume of 200 *µ*l.

#### 5.1.4. Imaging device setup

A PDMS sheet (1 × 1 cm, 250 *µ*m thick) was punched to create six through-holes (2 mm diameter, Cat. 21909-140, Miltex Inc.). This sheet was bonded to a glass-bottom dish (100 *µ*m thickness) using an oxygen plasma oven (Harrick Plasma Cleaner PDC-001). For further details on device setup and surface activation, refer to [59].

#### 5.1.5. 3D spheroid seeding

On the day of the experiment, the prepared PDMS wells were cooled on ice. A 2.5 *µ*l spheroid-collagen solution was added to each well, and the device was incubated at 37 ^∘^C and 5% CO_2_ for 45 min. To ensure suspension of spheroids during collagen polymerization, the device was inverted multiple times at 10, 15, and 20 min intervals. Post-polymerization, 1 ml DMEM or DMEM/F12 growth medium was added to the wells, and the plate was returned to the incubator for 2 h. After collagen matrix reorganization, spheroids were fixed with 4% PFA (Cat. SC281692, Santa Cruz) for 20 min, washed thrice with PBS, and transferred for imaging.

### 5.2. Imaging and data analysis

#### 5.2.1. Imaging of spheroid-embedded collagen matrices.

ECMs were imaged using reflectance confocal microscopy with a 405 nm laser, C-Apochromat 40×/1.20 W Corr M27 objective (Zeiss), and an LSM710 Zeiss confocal microscope. Typical image dimensions were 354.2 × 354.2 × 100 *µ*m with voxel sizes of 0.692 × 0.692 × 0.4 *µ*m (*x*, *y*, and *z*). Images represented maximum intensity *z*-projections of 15 *µ*m centered around the spheroid mid-plane.

### 5.3. Measurements

After performing the simulations for a range of target cell shape indices and for two different interfacial tensions, the following quantities were then measured. To quantify how the fiber network is remodeled, we compute the magnitude of the fiber displacement as a function of the distance from the center of mass of the spheroid. We also measure the fiber density as a function of the distance from the center of mass of the spheroid to further quantify the fiber remodeling extent by the spheroid. Finally, in terms of spatial fiber network reorganization, we compute the fiber orientation tensor using spherical coordinates. More specifically, the fiber orientation tensor *Ω*_*αβ*_ is given by

\begin{equation*} \Omega_{\alpha \beta} = \sum \frac{L_\mathrm{f}n_{\alpha\beta}}{L_\mathrm{tot}},\end{equation*}

where *α* and *β* denote the component in three dimensions, $L_\mathrm{f}$ denotes fiber length, $L_\mathrm{s}$ denotes the total system length, and $\hat{n}$ denotes the unit vector along the fiber axis. Should the fiber network be isotropically organized, $\Omega_{11} = \Omega_{22} = \Omega_{33} = 1/3$.

We explore how certain components of the fiber orientation tensor in spherical coordinates, such *Ω*_*rr*_, behaves on average as a function of distance from the center of mass of the spheroid. Moreover, as the fiber network is spatially remodeled, tension and compression will build up in the fiber network, particularly as the active linker springs contract to pull on the fiber network. We record the amount of tension/compression in the fiber network as the coupled system evolves in time. To understand how the active linker springs mediate the interaction between the spheroid and the fiber network, we also record the tension/compression in each of the linker springs over time.

Given that we have cellular-scale resolution of the spheroid, we will not only keep track of the overall spheroid shape index to determine how far from spherical it deviates, we will also study the shape index for the individual cells as a function of time. To quantify the amount of cell motion within the spheroid, we will compute displacements of the cell center of masses over time, which gives us a measure of the spheroid displacement. Finally, we also record the fraction of cells that lose two or more neighbors over time [19]. More fluid-like spheroids undergo more cellular rearrangements than solid-like ones.

## Data Availability

The data and code required to reproduce our analyses are available on Zenodo at https://doi.org/10.5281/zenodo.15656848.
